# Acoustic Performance Study of Fiber-Optic Acoustic Sensors Based on Fabry–Pérot Etalons with Different Q Factors

**DOI:** 10.3390/mi13010118

**Published:** 2022-01-12

**Authors:** Jiamin Chen, Chenyang Xue, Yongqiu Zheng, Jiandong Bai, Xinyu Zhao, Liyun Wu, Yuan Han

**Affiliations:** The State Key Laboratory of Dynamic Measurement Technology, North University of China, Taiyuan 030051, China; 18335160365@163.com (J.C.); xuechenyang@nuc.edu.cn (C.X.); jdbai@nuc.edu.cn (J.B.); zhaoxinyu_0803@163.com (X.Z.); wuliyun1211@126.com (L.W.); hanyuan96688@163.com (Y.H.)

**Keywords:** fiber-optic acoustic sensor, Fabry–Pérot etalon, quality factor, weak sound detection, high sound pressure detection, wide application range

## Abstract

The ideal development direction of the fiber-optic acoustic sensor (FOAS) is toward broadband, a high sensitivity and a large dynamic range. In order to further promote the acoustic detection potential of the Fabry–Pérot etalon (FPE)-based FOAS, it is of great significance to study the acoustic performance of the FOAS with the quality (Q) factor of FPE as the research objective. This is because the Q factor represents the storage capability and loss characteristic of the FPE. The three FOASs with different Q factors all achieve a broadband response from 20 Hz to 70 kHz with a flatness of ±2 dB, which is consistent with the theory that the frequency response of the FOAS is not affected by the Q factor. Moreover, the sensitivity of the FOAS is proportional to the Q factor. When the Q factor is $1.04\times 10^{6}$, the sensitivity of the FOAS is as high as 526.8 mV/Pa. Meanwhile, the minimum detectable sound pressure of $347.33\;\mu \mathrm{Pa}/{\mathrm{Hz}}^{1/2}\;$ is achieved. Furthermore, with a Q factor of $0.27\times 10^{6}$, the maximum detectable sound pressure and dynamic range are 152.32 dB and 107.2 dB, respectively, which is greatly improved compared with two other FOASs. Separately, the FOASs with different Q factors exhibit an excellent acoustic performance in weak sound detection and high sound pressure detection. Therefore, different acoustic detection requirements can be met by selecting the appropriate Q factor, which further broadens the application range and detection potential of FOASs.

## 1. Introduction

As a device of acoustic signal detection, the acoustic sensor is widely used in daily life and military, medical and aerospace fields, and has become an indispensable part of the development of modern society. Traditional electroacoustic sensors are the main force of commercial microphones because of their mature manufacturing technology [1]. Electroacoustic sensors are mainly divided into capacitive [2], piezoelectric and piezoresistive types, and the basic principle is based on the deformation of the membrane to sensing sound. Many membrane materials (zinc oxide [3], silicon nitride [4] and aluminum [5]) were implemented to increase the sensitivity, as well as several designs to lower the noise and flatten the frequency response. The use of membranes creates a trade-off between the sensor size and performance. For instance, while large-sized piezoelectric acoustic sensors possess a high sensitivity and signal-to-noise ratio, it is disadvantageous to sensor miniaturization that is urgently desired in practical applications, and the low resonant frequency of membranes result in a narrow frequency response of sensors. Furthermore, in order to adapt to the expansion of the acoustic detection field and the application requirements of the harsh environment, fiber-optic acoustic sensors (FOASs) with the advantages of a high sensitivity [6,7,8,9], immunity to electromagnetic interference [10,11,12], high temperature resistance and miniaturization [13,14] have been widely studied and applied in the fields of national defense security [15,16], medical diagnosis [17,18,19] and non-destructive testing [20,21]. At present, optical fiber [8] and membrane [22,23,24,25,26] are the most commonly used sound-sensitive element. The coupling of light and sound fields in FOASs based on them is indirect coupling. For example, the slight deformation of the optical fiber under the action of the sound field will affect the optical parameters of the optical fiber to realize the modulation of the light intensity by the sound field and to achieve acoustic detection. However, the sensitivity of FOAS is low because of the poor matching of acoustic impedance between the air and the fiber, and between the fiber cladding and the fiber core. A Fabry–Perot interferometer (FPI) FOAS is the representative of membrane-based FOASs [27,28,29]. The performances of the membrane determine that the FOAS with a greater sensitivity requires a thinner membrane. However, the resonance frequency of the thinner membrane is smaller, which limits the flat frequency response range of the FOAS, and the thinner membrane is easily broken when responding to acoustic signals with high sound pressure level, which further results in a small dynamic range. To achieve a higher sound pressure level detection, it is necessary to increase the thickness of the membrane, which will greatly reduce the sensitivity of the FOAS. Moreover, when the thickness of the membrane increases, the mechanical resonance frequency becomes larger, and the frequency response range of the FOAS should theoretically become larger. But research concentrating on high sound pressure signal detection rarely reports the flat frequency response range, and usually only tests individual frequency points, which indicates that the frequency response characteristics of the membrane-based FOAS under a high sound pressure detection are not ideal [30,31].

The acoustic sensitivity mechanism realized by using light beams to detect the slight change in the refractive index of the air medium caused by sound waves to realize the sound detection enables the FOAS to get rid of the limitation of the acoustic coupling material, and the acoustic response of the wideband and large dynamic range can be realized easily. Based on the above sound sensitivity mechanism, Fabry–Perot etalon (FPE)-based [32] and Mach–Zehnder interferometer [33] FOASs have been reported. Among them, the Mach–Zehnder interferometer membrane-free FOAS achieves a frequency response of 500 Hz to 20 kHz and a sensitivity of 77 mV/Pa. However, its sound-sensitive structure is the air cavity formed by the alignment and coupling of a pair of collimators (Thorlabs, 50-1550A-APC, Newton, NJ, USA), which has the disadvantages of a poor mechanical stability and large size. The membrane-free FOAS based on FPE adopts a rigid structure of all glass, there is no mechanical displacement of the cavity length during the sensing process and a wide and flat frequency response range can be achieved. Table 1 summarizes and compares these acoustic sensors mentioned above, listing their sensitive mechanism, sensitive elements and acoustic performance indicators, respectively. It can be seen that both capacitive and piezoelectric acoustic sensors achieve a large dynamic range, but the frequency response bandwidth is narrow and the sensitivity is low. A high sound detection sensitivity can be realized by the FOAS based on the optical fiber and membrane. However, they can only achieve a large bandwidth in water, and the bandwidth achieved in the air is average. Moreover, their dynamic range is not mentioned in the references. In addition, it has been reported that the frequency response characteristics of the FOAS based on the membrane, which can realize a high sound pressure detection and high sensitivity, are not ideal. In contrast, a membrane-free FOAS based on FPE can overcome the limitations of these acoustic sensors, and a wideband, high sensitivity and large dynamic range is achieved. Meanwhile, the acoustic performance of the membrane-free FOAS based on the Mach–Zehnder interferometer may need further optimization.

Recently, our research group proposed the FOAS based on a high quality factor resonance effect using FPE, which employs phase modulation spectroscopy and resonance frequency tracking and locking technology to perform acoustic signal demodulation, and the high sensitivity, large dynamic range and application flexibility can be realized [34]. Besides, the rigid structure and size of the FPE determine that it can withstand an ultra-high sound pressure of 236.8 dB, indicating that the high sound pressure detection performance of the FOAS can be further greatly improved. Therefore, it is necessary to study the acoustic performance of FOAS deeply. As the core acoustic sensing element of the FOAS, the characteristics of the FPE will directly affect the acoustic performance of the FOAS, and the quality (Q) factor is an index to evaluate the performance of the FPE, which represents the storage capability and loss characteristic of the FPE to energy coupled into the cavity, which can be defined as Equation (1). It can be seen that the higher the Q factor, the stronger the energy storage capacity of the FPE, the smaller the loss of FPE and the longer the lifetime of photons in the FPE.

(1) $$Q=2\pi \nu \frac{\varepsilon }{P}$$

where ν is the oscillation frequency of the electromagnetic field in FPE, ε is the total energy stored in the FPE and P is the energy lost per unit time.

In this paper, three FPEs with different Q factors are selected as the core acoustic sensing elements of the FOASs, of which, the acoustic performances are theoretically analyzed and experimentally tested, respectively. It can be obtained from the experimental results that the frequency response range of the FOASs based on the FPEs with three different Q factors remains unchanged, which is from 20 Hz to 70 kHz with a flatness of ±2 dB. The sensitivity of the FOAS is proportional to the Q factor of the FPE. An ultra-high sensitivity of 526.8 mV/Pa is achieved when the Q factor of the FPE is $1.04\times 10^{6}$, and the minimum detectable sound pressure is $347.33\;\mu \mathrm{Pa}/{\mathrm{Hz}}^{1/2}$ at the same time. Moreover, it is more meaningful that the maximum detectable sound pressure increases by 6.73 dB from 147.53 dB to 152.32 dB when the Q factor of the FPE is reduced from $1.04\times 10^{6}$ to $0.27\times 10^{6}$. Meanwhile, the dynamic range of the FOAS also increases by 0.6 dB from 106.6 dB to 107.2 dB. By means of the study on the acoustic performance of the three FOASs, it is found that the FOASs exhibit an excellent acoustic performance of weak sound detection and high sound pressure detection, respectively, while maintaining the broadband response. For example, the turbulent noise of gunpowder gas at the tail of the nozzle during rocket launch is generally approximately 135 dB, and the high noise can reach 145–155 dB. Therefore, the proposed FOAS based on the FPE with low-Q can be used to monitor the noise of the rocket. In terms of human acoustics, the frequency of the sound signals generated by human physiological activities is mainly concentrated in the low frequency band, with a frequency not exceeding 2 kHz, and acoustic sensors with a high sensitivity, miniaturization, multi-point measurement and easy networking are required. The proposed FOAS based on the FPE with high-Q just meets this requirement. As a result, the test requirements of different acoustic detection fields can be met by selecting the appropriate Q factor. As a result, the application range and sound detection potential of the FOASs have been greatly improved.

## 2. Theoretical Analysis and Simulation

**Figure 1:**
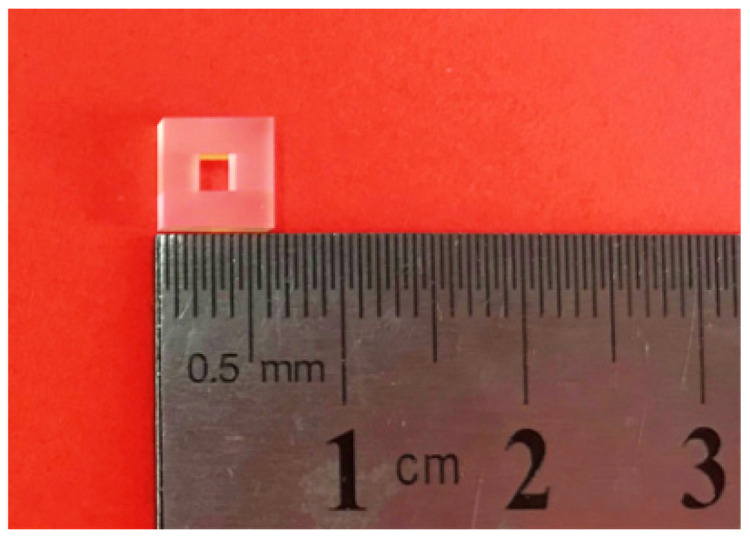
The photograph of the used FPE (Fabry–Pérot etalon).

According to the transfer function of the FPE [35], which is Equation (1), the characteristics of the FPE are determined by the mirror reflectivity and cavity length. In fact, the surface figure of the mirror and the parallelism between the mirrors also affect the characteristics of the FPE. Here, FPE adopts fully rigid ULE zero-expansion glass and is batch-fabricated by optical contact technology. The photograph of the used FPE is shown in Figure 1, of which, the cavity length and surface figure are 2 mm and less than λ/20, respectively, and the parallelism between the mirrors is also the same. Thus, the FPE with a different mirror reflectivity will exhibit different characteristics in accordance with Equation (2).

(2) $$T=\frac{I_{trans}}{I_{0}}=\frac{1}{1+\frac{4R{sin}^{2}(q/2)}{{\left(1-R\right)}^{2}}},\;q=4\pi nd/\lambda ,$$

where $I_{trans}$ is the transmission intensity, $I_{0}$ is the input intensity, R is the mirror reflectivity and q is the round trip phase shift, which depends on the laser wavelength λ, the mirror distance d and the refractive index of the medium between the cavity mirrors n.

On the basis of Equation (2), the full width at half maximum (FWHM) of the transmission curve expressed by the phase can be deduced as

(3) $$FWHM_{\Delta q}=4arcsin\frac{1-R}{2\sqrt{R}}.$$

Then, the FWHM expressed in frequency is written as

(4) $$FWHM_{\Delta f}=\frac{c}{\pi nd}arcsin\frac{1-R}{2\sqrt{R}}.$$

Therefore, the Q factor of FPE can also be expressed as

(5) $$Q=\frac{f_{laser}}{FWHM_{\Delta f}}=\frac{f_{laser}\pi nd}{carcsin\frac{1-R}{2\sqrt{R}}},$$

where $f_{laser}$ is the central frequency of the laser and Q is the *Q* factor of the FPE.

**Figure 2:**
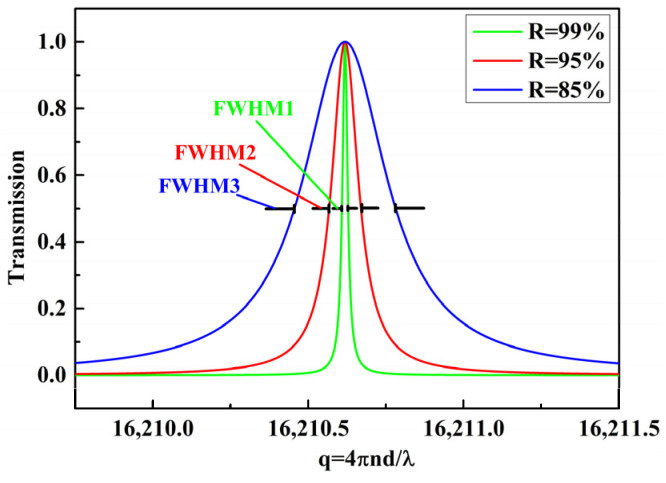
The transmission curves of the FPEs with mirror reflectivity of 99%, 95% and 85%.

The transmission characteristics of the FPEs with a mirror reflectivity of 99%, 95% and 85% are simulated by means of Equation (2). The obtained transmission curves are shown in Figure 2. It can be observed that, when the other parameters of FPE are the same, the FWHM1 is the narrowest, the FWHM2 is second and the FWHM3 is the largest. Moreover, according to Equation (5), the smaller the FWHM, the greater the Q factor. Therefore, the acoustic performance of FOASs based on FPEs with different Q factors can be verified by using the three FPEs with different reflectivity.

**Figure 3:**
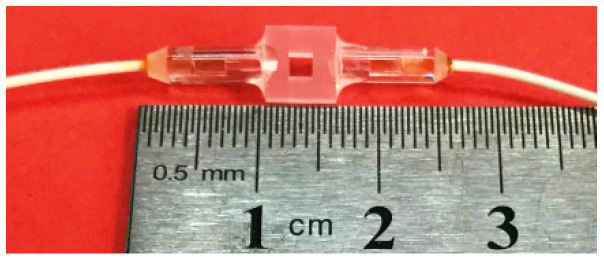
Photograph of the all-solid-state integrated fiber-optic acoustic sensor (FOAS).

**Figure 4:**
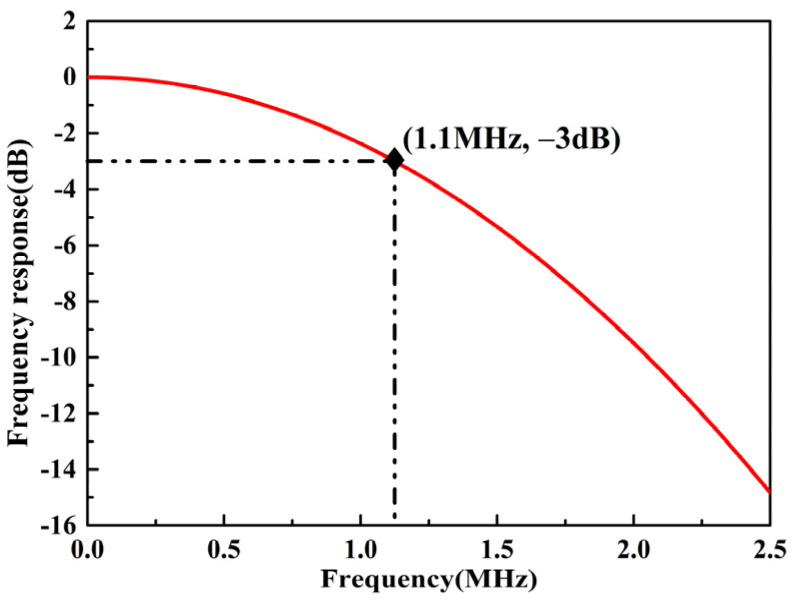
The simulated frequency response curve of the FOAS.

The FPE-based FOAS is an all-solid-state integrated FOAS, shown in Figure 3, which is realized by coupling and aligning two optical fiber collimators with the FPE through a five-dimensional electric alignment platform. The FOAS uses the change in the air refractive index caused by the change in the air molecule density between the FPE mirrors due to the disturbance of the sound waves in order to detect the acoustic signals. Due to the fact that the FPE is a rigid structure, the FOAS is not affected by the mechanical resonance frequency, and a flat broadband frequency response can be obtained. The upper limit of the detection frequency of FOAS depends on the diameter of the laser beam. The reason is that the frequency of sound waves increases and the wavelength becomes shorter. When the wavelength of the acoustic wave is as short as it is close to the diameter of the laser beam, the maximum and minimum values of sound pressure will appear in the laser beam at the same time, making the FOAS unable to output acoustic signals. The normalized response of the FPE-based FOAS can be calculated by the one-dimensional convolution of the Gaussian beam function and each frequency component, as shown in Equation (6) [36].

(6) $$p_{m}\left(k\right)=\int_{-\infty }^{\infty }p_{s}\left(k\right)sin(k\left(x-x’\right))\cdot exp(-\frac{2x^{2}}{\omega_{0}{}^{2}})\cdot dx’,$$

where k is an integer, $p_{s}\left(k\right)$ denotes the pressure amplitude at the location of the sensor for each frequency component, x is the wavelength of the sound wave, $\omega_{0}$ is the beam radius and $p_{m}\left(k\right)$ is the corresponding measured amplitude. Since the beam diameter of the fiber-optic collimator used in the FOAS is 300 μm, the frequency response curve of the FOAS can be simulated as shown in Figure 4, of which, the −3 dB frequency bandwidth can reach 1.1 MHz. As a result, the frequency response of the FOASs is theoretically independent of the Q factor of the FPE.

**Figure 5:**
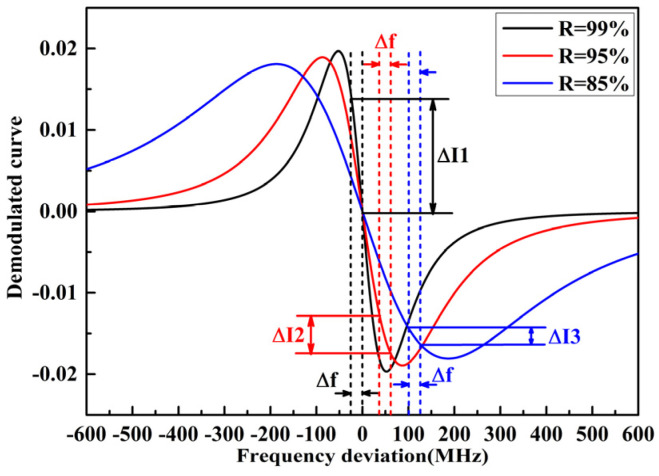
The simulation demodulated curves of the FPE with different reflectivity.

In the sensing process of the FOAS, the change in the air refractive index caused by the sound pressure results in a shift of the resonance frequency, which is proportional to the change in sound pressure. Since the frequency difference generated by the resonance frequency shift of the FPE is very weak, it is difficult to be measured directly. Phase modulation spectroscopy technology is used as the acoustic demodulation method for the detection of acoustic signals. The weak frequency difference signal can be amplified and extracted by means of phase modulation and lock-in amplification technology. Then, according to the correspondence relationship between the resonance frequency shift and the demodulated curve, the demodulated curve is used as an error signal to feedback and control the laser so that the laser frequency can track and be locked at the resonance frequency. After frequency locking, the effect of the acoustic signals on the resonance frequency corresponds to the offset of the locked demodulated curve relating to the null point. The working principle of the light field and electric field in the detection process of phase modulation spectroscopy technology has been analyzed in detail in our previous work. According to Equation (5) of Reference [34], the demodulated curves of FPE with different reflectivity can be simulated, as shown in Figure 5. Figure 5 illustrates that the greater the reflectivity of the FPE, the greater the slope of the demodulated curve. For a given resonance frequency offset, the amount of change in the linear amplitude of the demodulated curve, that is, the sensitivity of the FOAS, depends on the slope of the demodulated curve. Therefore, the sensitivity of the FOAS based on FPE with high reflectivity will be greater.

**Figure 6:**
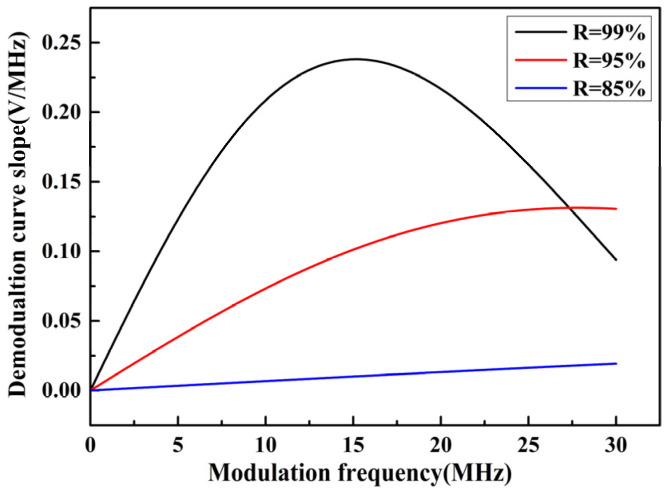
The simulation slope change characteristics of the FOASs under different Q factors.

With the phase modulation spectroscopy technology, the slope of the demodulated curve is connected with the phase modulation frequency as well. In light of the expressions of the output demodulated signal and its slope under sine wave modulation, the slope change characteristics of the FOAS based on the FPE with three different Q factors under different modulation frequencies, that is, the sensitivity change characteristics, can be obtained, as shown in Figure 6. As can be seen from Figure 6, with the decrease in reflectivity, the slope of the demodulated curve becomes smaller on the whole, indicating that the sensitivity of the FOAS will become smaller. Besides, the dynamic range is determined by the minimum and maximum detectable sound pressure. The minimum detectable sound pressure is related to the sensitivity. The greater the sensitivity, the smaller the minimum detectable sound pressure. The maximum detectable sound pressure is related to the sensitivity and linear amplitude of the demodulated curve. A smaller sensitivity and a larger amplitude of the demodulated curve can lead to a larger maximum detectable sound pressure. Although the sensitivity characteristics of the FOAS can be obtained via theoretical analysis, since the linear amplitude of the demodulated curve changes randomly, the maximum detectable sound pressure performance of the FOAS needs to be further measured experimentally.

## 3. Experiment Measurement and Discussion

**Figure 7:**
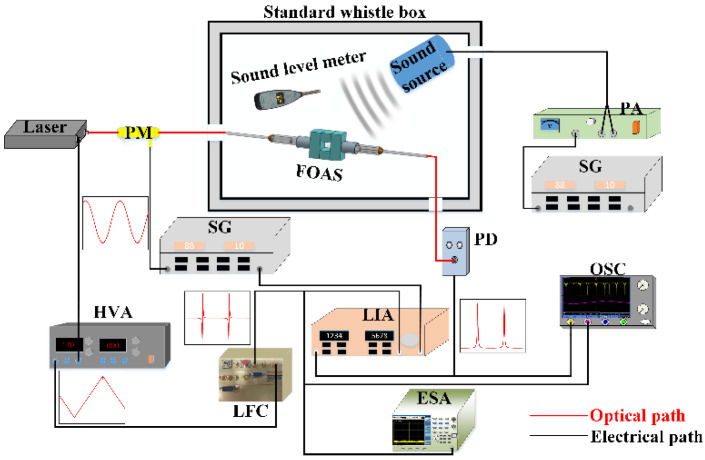
The acoustic performance test system diagram of FOAS. The optical path is drawn in red and the electrical path is drawn in black.

**Figure 8:**
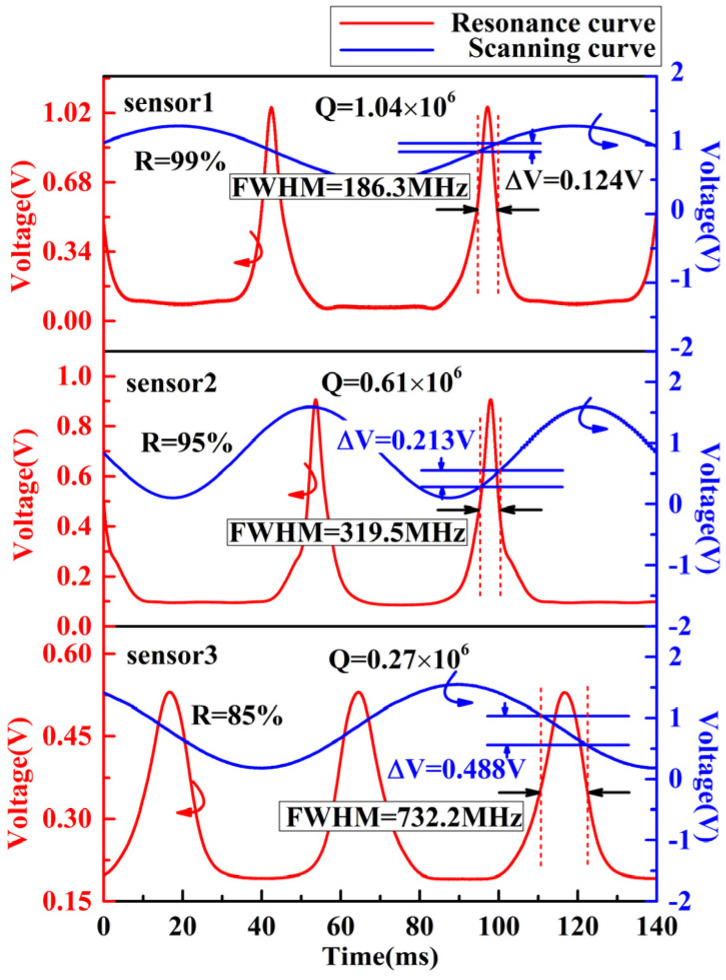
The resonance spectrum of the used FPEs with reflectivity of 99%, 95% and 85%.

In order to test the characteristics of the three FPEs and the acoustic performance of FOASs, the acoustic performance test system is set up as shown in Figure 7. The triangular wave signal output by the lock-frequency controller (LFC) is amplified 100 times by the high voltage amplifier (HVA, YG2009A-350V-2bip) and then applied to the PZT scanning port of the laser (NKT E15) to sweep the laser frequency, so that the resonance spectrum of FPE can be obtained on the oscilloscope (OSC, MDO4104C). Figure 8 is the resonance spectrum of the used FPEs with reflectivity of 99%, 95% and 85%, of which, the sampling rate is 50 kHz. According to the relationship between the resonance spectrum and the scan curve, the FWHM of the resonance spectrum can be obtained from the voltage difference in the scan curve corresponding to its half height and the PZT tuning coefficient of the laser that is 15 MHz/V. Due to the fact that the voltage of the scanning curve observed on the oscilloscope is 100 times lower than the scanning voltage applied to the laser, the calculation formula for the FWHM of the resonance spectrum is FWHM=ΔV×15 MHz/V×100. Therefore, the Q factors of sensor 1, sensor 2 and sensor 3 can be calculated as $1.04\times 10^{6}$, $0.61\times 10^{6}$ and $0.27\times 10^{6}$.

Then, the above-mentioned experimental system is used to test the acoustic performance of the FOASs. In order to eliminate environmental noise, the FOAS, sound level meter (AWA5661) and sound source (HF-6522) are placed in a standard whistle box (WDF-XYX001). The FOAS and the calibrated reference sound level meter are placed symmetrically and are located equidistant to the sound source. The FOAS receives the sound signals from the sound source, which is driven by a power amplifier (PA, AWA5871) and a signal generator (SG, MFG-2260MRA). A frequency sweep signal from 20 Hz to 100 kHz can be generated by the SG and applied to the PA. Moreover, the optical detection process of acoustic signals is that a narrow linewidth laser with a central wavelength of 1550 nm is connected to the phase modulator (PM, GC15PMPL7813), which illuminates the FOAS. The transmitted light is received by the photodetector (PD, Newport 2053-FC-M), and the converted electric signal is demodulated using the lock-in amplifier (LIA, SR844). The demodulated signal is used as an error signal for the feedback control of the laser frequency in order to achieve frequency locking through the lock-frequency controller. After frequency locking, the effect of the acoustic signal on the resonance frequency corresponds to the offset of the demodulation curve relative to zero; thus, intuitive acoustic detection is to be realized.

**Figure 9:**
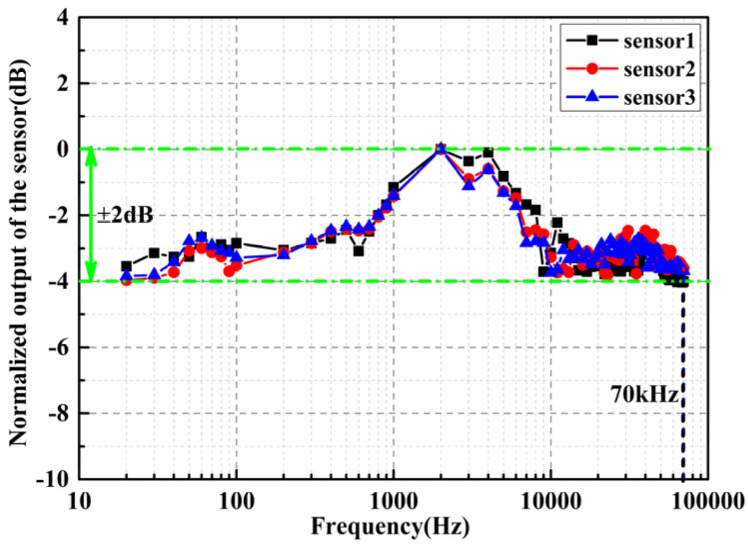
The frequency response curve of the three FOASs.

By applying acoustic signals with a constant amplitude and frequency ranging from 20 Hz to 100 kHz to the FOAS, the frequency domain response of the FOAS to the acoustic signals can be directly collected from the electrical spectrum analyzer (ESA, N9030A). The collected frequency response amplitudes are normalized to dB to obtain the frequency response characteristic of the FOAS. Figure 9 shows the frequency response curves of the three FOASs, from which, the frequency response from 20 Hz to 70 kHz with a flatness of ±2 dB is achieved. Moreover, the sampling rate of the response spectrum from 20 Hz to 70 kHz collected from ESA is 546.99 Hz (frequency range from 20 Hz to 90 Hz), 4955.45 Hz (frequency range from 100 Hz to 900 Hz) and 88,584.07 Hz (frequency range from 1 kHz to 70 kHz), respectively. Here, the three sensors are the FOASs with Q factor of $1.04\times 10^{6}$, $0.61\times 10^{6}$ and $0.27\times 10^{6}$, respectively. The experimental results fully prove the correctness of the theory that the frequency response of the FOAS based on the rigid FPE is only related to the diameter of the incident laser beam. This wideband response covers the ultrasonic frequency range from 20 kHz to 70 kHz, and they can be used as an ultrasonic receiver in ultrasonic nondestructive testing technology. This method of industrial non-destructive testing through FOASs has the advantages of a resonance-free response and much reduced dead zone compared to the method of measuring vibration characteristics through a vibration accelerometer. The difference between the measured frequency response range and the theoretically measurable frequency bandwidth of the FOAS is mainly due to the limitation of the feedback control mode of the laser, the response speed of the lock-frequency controller and the bandwidth of some other electrical components in the demodulation system. In the subsequent work, a full closed loop detection method based on phase modulation would be adopted. The center frequency of the laser would be locked at the resonance frequency of the FPE in real time, and the acoustic signal can be detected by reading out the feedback error signal controlling the laser, which can greatly expand the frequency response bandwidth.

**Figure 10:**
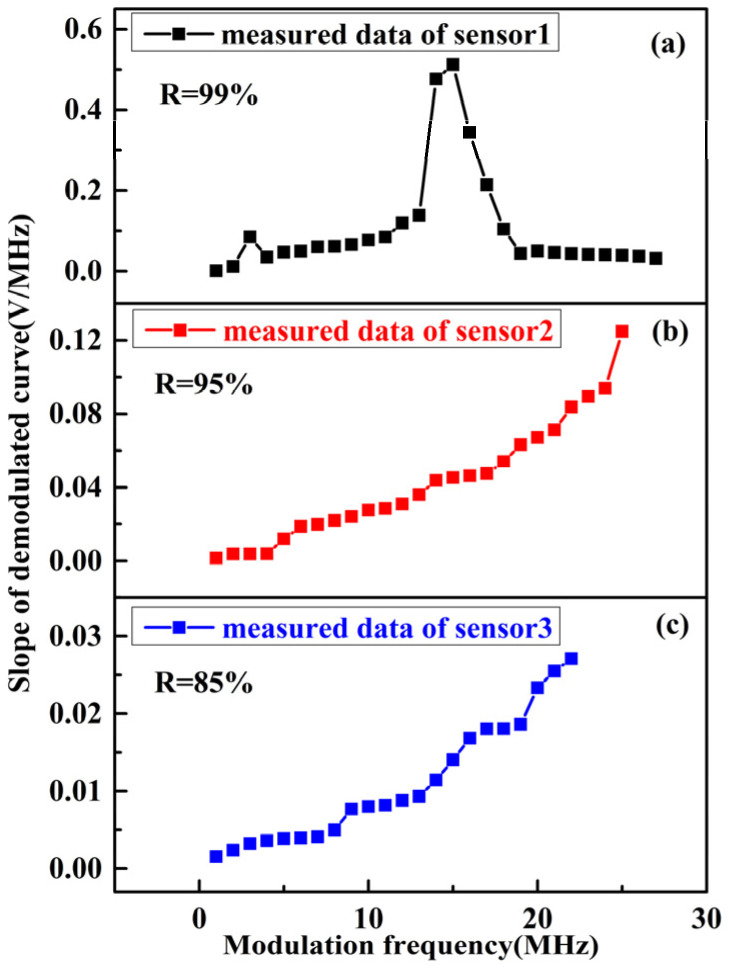
The measured changes in the slope of the FPE demodulated curve with different Q factors under different modulation frequencies: (**a**) R is 99%; (**b**) R is 95%; (**c**) R is 85%.

**Figure 11:**
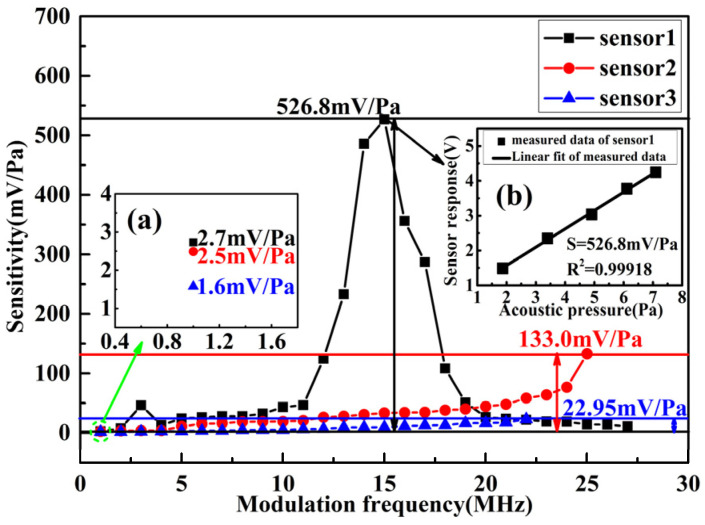
The sensitivity characteristics of the three FOASs when the modulation frequency changes from 1 MHz to 27 MHz. The inset (**a**) shows the sensitivities of the three sensors at a modulation frequency of 1 MHz, and the inset (**b**) shows the sensitivity test data and linear fitting result of sensor 1 at the modulation frequency of 15 MHz.

The SG generates signals, of which, the frequency is fixed at 1 kHz and the amplitude is changed from 1 V to 5 V with a step of 1 V in order to drive the sound source. The response voltage of the FOAS and the sound pressure of the sound level meter under acoustic signals with different amplitudes are collected, respectively. After linear fitting of the response voltage and sound pressure, the fitting slope is the sensitivity of the FOAS. Besides, according to the above analysis, the FOAS exhibits different demodulated curve slopes and sensitivities under different modulation frequencies. Here, the demodulated characteristics and sensitivity of FOASs are also tested under the condition that the amplitude of the modulation signal is fixed as 10 Vpp and the frequency changes from 1 MHz to 27 MHz with a step of 1 MHz. The sampling rate of the measured demodulated curves of FOASs is 500 kHz, and, in the sensitivity test process, the sampling rate of the response results of FOASs to different sound pressure level signals is also 500 kHz. The test results are shown in Figure 10 and Figure 11. Compared with Figure 6, it can be found that their change trend in the experimental results is consistent with the theoretical analysis.

Moreover, the sensitivity of the FOAS is also associated with the Q factor of the FPE. The higher the Q factor, the greater the sensitivity that can be obtained. As can be seen from Figure 11, the average sensitivity of sensor 1 is the highest, followed by sensor 2, and sensor 3 is the lowest in the process of changing the modulation frequency. The three FOASs achieve maximum sensitivities of 526.8 mV/Pa, 133.0 mV/Pa and 22.95 mV/Pa, respectively. Another performance index corresponding to sensitivity is the minimum detectable sound pressure. The greater the sensitivity, the stronger the ability of the FOAS to pick up weak sound signals; that is to say, the minimum detectable sound pressure (MDP) is smaller. The equation for calculating the MDP is defined as

(7) $$MDP=\frac{P_{in}}{10^{SNR/20}\times \sqrt{RBW}},$$

where $P_{in}$ is the input sound pressure of the sound source and SNR and RBW are the signal-to-noise ratio and resolution bandwidth of the response spectrogram of FOAS at a 1 kHz acoustic signal.

**Figure 12:**
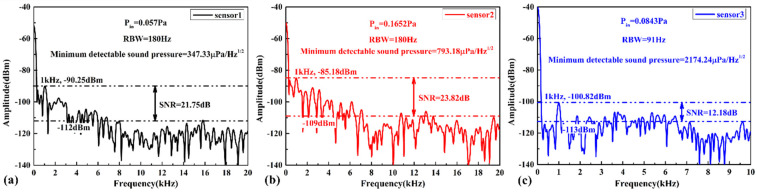
Response spectrograms of (**a**) sensor 1, (**b**) sensor 2 and (**c**) sensor 3 at 1 kHz acoustic signal when the maximum sensitivity is obtained.

When the maximum sensitivities of the three sensors are obtained, the 1 kHz signals with the minimum sound pressure that the sensors can respond to are tested, and the response spectrograms are shown in Figure 12. According to Equation (7), the minimum detectable sound pressures can be obtained as $347.33\;\mu \mathrm{Pa}/{\mathrm{Hz}}^{1/2}$, $793.18\;\mu \mathrm{Pa}/{\mathrm{Hz}}^{1/2}$ and $2174.24\;\mu \mathrm{Pa}/{\mathrm{Hz}}^{1/2}$, corresponding to the minimum detectable sound pressure levels of 24.79 dB, 31.97 dB and 40.73 dB, separately. The calculated results fully indicate that sensor 1 with a high Q factor has the strongest ability to detect weak acoustic signals.

**Figure 13:**
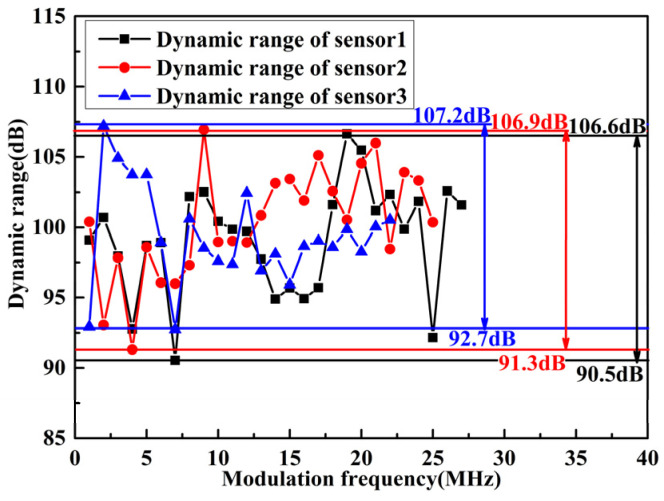
The dynamic range of the FOASs based on the FPEs with different Q factors under the modulation frequency from 1 MHz to 27 MHz.

**Figure 14:**
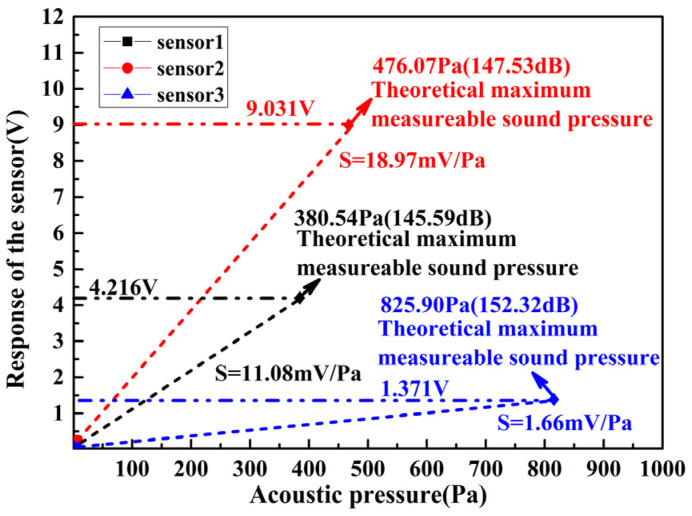
The maximum detectable sound pressure that can be achieved by the three FOASs.

The dynamic range of the FOAS is also one of its important performance indicators, and is determined by the maximum and minimum detectable sound pressure, respectively. As demonstrated in Figure 13, the dynamic range of the three sensors under a modulation frequency from 1 MHz to 27 MHz are tested. We can conclude that the three sensors have achieved a large dynamic range greater than 90 dB. Moreover, as the Q factor decreases, the dynamic range increases. In particular, the larger dynamic range of 107.2 dB is achieved by sensor 3, which is better than sensor 1 and sensor 2. This is mainly due to the fact that sensor 3 exhibits a higher maximum detectable sound pressure. The maximum detectable sound pressure is determined by the sensitivity and the amplitude of the linear region of the demodulated curve. Figure 14 exhibits the maximum detectable sound pressure that can be achieved by the three sensors under different modulation frequencies. Among them, with a Q factor of $0.27\times 10^{6}$, the FOAS achieves a maximum sound pressure detection of 152.32 dB, which is 4.79 dB and 6.73 dB higher than the FOASs based on the FPEs with Q factors of $0.61\times 10^{6}$ and $1.04\times 10^{6}$, respectively.

**Figure 15:**
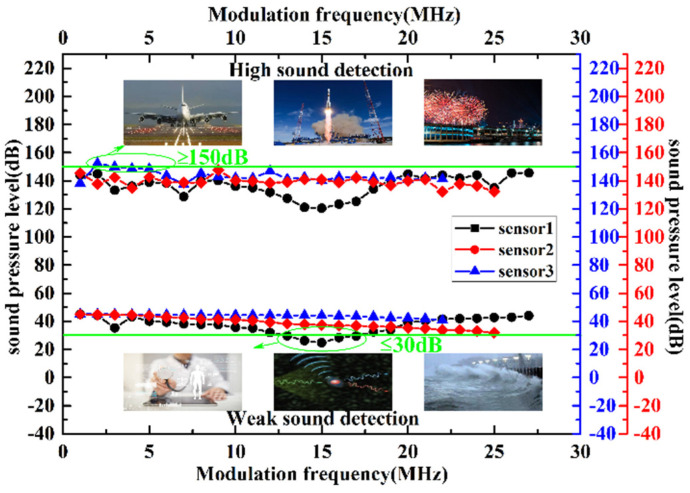
The maximum and minimum detectable sound pressures of the three FOASs at different modulation frequencies.

The maximum and minimum detectable sound pressures of the three sensors at different modulation frequencies are shown in Figure 15. It can be seen from the detectable sound pressure range of the proposed FOAS that it can be used to monitor the bridge noise, of which, the frequency range is from 20 Hz to 200 Hz and the sound pressure range is from 60 dB to 95 dB. Here, the fiber optic sound sensor is used as a noise sensor. In order to more intuitively describe the performance differences in the three sensors, the optimal performance that they can achieve is compared in Table 2. It is worth mentioning that the minimum detectable sound pressure achieved by sensor 1 is less than 30 dB, which can be used in the field of weak sound detection, such as medical diagnosis, photoacoustic imaging and natural disaster warning. Moreover, sensor 3 achieves maximum detectable sound pressure level greater than 150 dB, which determines that it can be widely used in the noise monitoring of rocket launches and fireworks displays. More importantly, the above mentioned advantages illustrate that, for a variety of acoustic detection fields, a suitable FOAS can be found as long as the appropriate Q factor is selected, which provides a promising and attractive development direction for the FOAS based on the FPE.

## 4. Conclusions

As the key sensing element of the FOASs, FPEs with different Q factors will make the FOASs have different acoustic performances. The related theoretical analysis and experimental measurements have been comprehensively studied in this paper. Since the frequency response of the FOAS depends on the diameter of the incident beam, three FOASs all achieve a broadband response from 20 Hz to 70 kHz with a flatness of ±2 dB. The sensitivity of the FOAS is proportional to the quality factor of FPE, and the ultra-high sensitivity of 526.8 mV/Pa is achieved when the quality factor of FPE is $1.04\times 10^{6}$. Moreover, the FOAS based on the FPE with a quality factor of $0.27\times 10^{6}$ achieves an ultra-high detectable sound pressure of 152.32 dB, which is 4.79 dB and 6.73 dB higher than the FOASs based on the FPEs with quality factors of $0.61\times 10^{6}$ and $1.04\times 10^{6}$, respectively. On the basis of this, the dynamic range of the FOAS with a quality factor of $0.27\times 10^{6}$ has also been improved, so that the large dynamic range of 107.2 dB is realized. In practical application, the excellent acoustic performance that the three FOASs exhibit proves that they can be flexibly applied to different acoustic detection fields. At the same time, the acoustic detection potential of the FOAS has been greatly excited, which provides a strong support for the further development of fiber-optic acoustic sensing technology.

## Figures and Tables

**Table 1:** Summary and comparison of the performance of acoustic sensors.

Author (Year)	Sensing Scheme/Element	Bandwidth	Sensitivity/MDP/NEP	Dynamic Range	Ref.
B&K4189 N/A	Capactive/ stainless-steel diaphragm	6.3–20 kHz	50 mV/Pa	14.6–146 dB	[1]
Ashish Kumar (2019)	Piezoelectric/ ZnO	12 Hz–22 kHz	80 uV/Pa	100–180 dB	[3]
Wonuk Jo (2013)	Fabry–Perot interferometric/ photonic-crystal membrane	100 Hz–30 kHz	2.6 uPa/Hz^1/2^	N/A ^1^	[26]
Xiuxin Wang (2019)	Fabry–Perot interferometric/ microfiber	35 MHz	18 Pa	N/A ^1^	[8]
Pingjie Fan (2020)	Michelson interferometric/ gold diaphragm	0.8 to 250 Hz	−130.6 dB re 1 rad/Pa@100 Hz	N/A ^1^	[25]
Shuai Wang (2019)	Fabry–Perot interferometric/ PET film	N/A ^1^	37.1 nm/Pa	62.2–92.4 dB	[30]
Jiamin Chen (2020)	Fabry–Perot etalon/ Membrane-free	20 Hz–70 kHz	177.6 mV/Pa 530 uPa/Hz^1/2^	29.4–144.78 dB	[31]
Wenhua Zhu (2020)	Mach–Zehnder interferometer/membrane-free	500 Hz to 20 kHz	77 mV/Pa	N/A^1^	[32]

^1^ N/A = data not available.

**Table 2:** Optimal performance comparison of the three sensors.

Sensor	Frequency Range (Hz)	Maximum Sensitivity (mV/Pa)	Minimum Detectable Sound Pressure (Level) ($\mu Pa/Hz^{1/2}(\mathrm{dB}))$	Maximum Dynamic Range (dB)	Maximum Detectable Sound Pressure (Level) (Pa (dB))
sensor 1	20–70 k	526.8	347.33 (24.79)	106.6	380.54 (145.59)
sensor 2	20–70 k	133.0	793.18 (31.97)	106.9	476.07 (147.53)
sensor 3	20–70 k	22.95	2174.24 (40.73)	107.2	825.90 (152.32)
